# The Role of the Sol-Gel Synthesis Process in the Biomedical Field and Its Use to Enhance the Performance of Bioabsorbable Magnesium Implants

**DOI:** 10.3390/gels8070426

**Published:** 2022-07-07

**Authors:** Juan Pablo Fernández-Hernán, Belén Torres, Antonio Julio López, Joaquín Rams

**Affiliations:** Área de Ciencia e Ingeniería de Materiales, ESCET, Universidad Rey Juan Carlos, C/Tulipán s/n, 28933 Móstoles, Spain; belen.torres@urjc.es (B.T.); antoniojulio.lopez@urjc.es (A.J.L.); joaquin.rams@urjc.es (J.R.)

**Keywords:** sol-gel, biomaterials, coatings, scaffolds, matrices, nanoparticles

## Abstract

In the present day, the increment in life expectancy has led to the necessity of developing new biomaterials for the restoration or substitution of damaged organs that have lost their functionalities. Among all the research about biomaterials, this review paper aimed to expose the main possibilities that the sol-gel synthesis method can provide for the fabrication of materials with interest in the biomedical field, more specifically, when this synthesis method is used to improve the biological properties of different magnesium alloys used as biomaterials. The sol-gel method has been widely studied and used to generate ceramic materials for a wide range of purposes during the last fifty years. Focused on biomedical research, the sol-gel synthesis method allows the generation of different kinds of biomaterials with diverse morphologies and a high potential for the biocompatibility improvement of a wide range of materials commonly used in the biomedical field such as metallic implants, as well as for the generation of drug delivery systems or interesting biomaterials for new tissue engineering therapies.

## 1. Introduction

**Figure 1:**
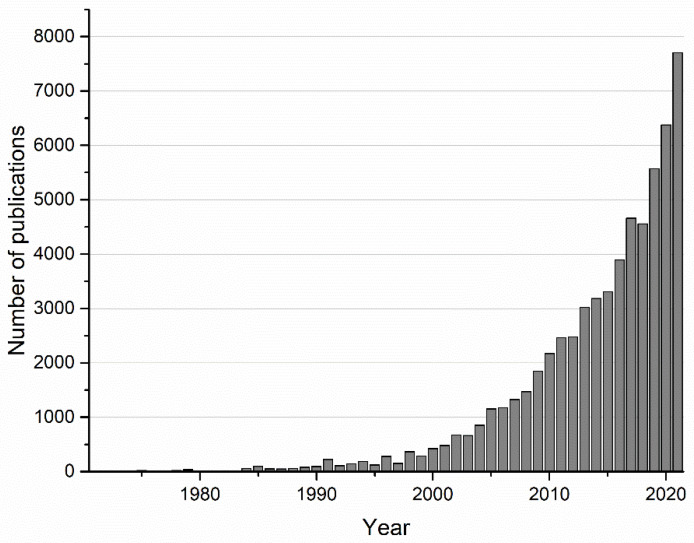
Number of publications related to biomaterials and biocompatibility from 1970 to 2021 based on the Science Direct search database.

Over the last decades, the available scientific information and interest in biomaterials have increased exponentially, especially from the year 2010 onwards, as can be seen in Figure 1, where the number of publications per year related to biomaterials, based on the Science Direct database, is shown. The increment in life expectancy and the subsequent emergence of diseases related to ageing lead to the necessity of developing new biomaterials for the manufacture of devices intended to be implanted in the body to repair or substitute damaged or lost tissues. The selection of a specific material depends on the intended application, considering the material properties. In the case of biomaterials, in addition to their mechanical, chemical, and physical properties, biological properties must be considered in terms of their interactions with biological environments. Thus, from the 1980s until today, different generations of biomaterials have been developed and classified depending on their biological properties [1,2,3].

First-generation biomaterials are characterized by their non-toxicity and bioinert behavior once implanted in a biological environment. After the implantation, these materials aim to obtain a minimum immune response. However, the body reacts by generating a fibrous capsule around the implant, isolating them from the surrounding tissues, to tolerate the presence of the implanted material. However, the formation of a fibrous capsule can lead to collateral problems. The most important issue, especially in bone restoration or fixation implants, is the low adhesion between the bone-implant interfaces. Without a good osseointegration level, the implant is likely to fail, and it is necessary to remove it from the body with a new surgery. Moreover, the presence of a fibrous capsule could result in a deficient blood supply to the surrounding tissues, harming them and increasing the probability of bacterial colonization of the implant, because the lack of blood flow can prevent the white cells from reaching and eliminating the bacteria present on the surface of the implant. Some common materials that could be classified as first-generation biomaterials are stainless steel (316L), titanium, and Cr-Co-Mo alloys.

Contrary to first-generation, second-generation biomaterials are not bioinert but bioactive, which means that once implanted in the body, these biomaterials can interact with the biological medium, causing a positive response from the tissues surrounding them. Thus, in the case of implants in contact with bone tissue, the bone cells can grow and spread on the surface of the implant, achieving improved osseointegration and decreasing the probability of the failure of the implant due to a solid bone–implant bonding. Another characteristic of second-generation biomaterials is their ability to dissolve in biological environments. Thus, once implanted, these biomaterials start to dissolve and stimulate cellular growth, being gradually substituted by natural tissue. Moreover, the by-products of the degradation process must be easy to eliminate, and not cause aggressive adverse responses from the surrounding tissues. The biodegradation implies that, in the case of bone fracture treatments, a second surgery to remove the implant after the healing process is no longer necessary, because the implant is gradually absorbed and substituted by natural bone tissue. Common biomaterials belonging to the second generation are hydroxyapatite (HAp) and bio-glasses (BG).

Third-generation biomaterials are characterized not only by their bioactive and biodegradation properties but by their ability to cause specific cellular responses at a molecular level, which means, for example, the expression of specific genes that cause cell growth and differentiation to restore specific damaged tissues. These materials are used in tissue engineering to create living tissues both in vitro and in vivo. In vitro living tissues can be grown on scaffolds made out of third-generation biomaterials and then implanted in the body to replace the damaged or lost tissues. After the implantation, the scaffold is meant to be reabsorbed and substituted by the natural tissue. In vivo treatment is based on the use of third-generation biomaterials in the state of powders or particles, which can stimulate cellular growth and differentiation to achieve in situ tissue regeneration.

From the year 2016 onwards new fourth-generation biomaterials were proposed to be developed. These biomaterials would be characterized by their ability for handling and monitor the cellular bioelectric signals to control the cell behavior [4]. Biomaterials belonging to the fourth generation are carbon-based materials and conductive polymers. Table 1 summarizes the different generations of biomaterials.

The belonging-to-a-specific-biomaterial generation can be modified. There are several procedures and methods in material science and engineering that can be applied to modify the initial properties of a material. For example, the bioinert properties of a first-generation biomaterial can be modified through different methods such as coating their surface with bioactive coatings or by changing their surface composition and microstructure, providing new bioactive properties and promoting the biomaterial from the first to the second generation.

At this point, the sol-gel synthesis method generates interest. The sol-gel synthesis process to obtain ceramic materials was discovered and used for the first time in the 19th century when J. Ebelmen produced the first silica gels in 1846. However, the sol-gel process did not raise interest until the 20th century, when it experienced great advances, especially from 1960 onwards. Nowadays, it is still being used for the manufacture of cutting-edge biomaterials with a wide range of applications such as biocompatibility improvement of implants and bone-fracture treatment materials [5,6,7,8,9], drug delivery systems [10,11,12], research on bioartificial organs, and tissue engineering therapies [13,14]. Sol-gel is a versatile process that, combined with an appropriate coating method, can be used to generate coatings deposited on non-bioactive materials to change their properties, increasing their biocompatibility, biocorrosion protection, or antibacterial properties.

**Figure 2:**
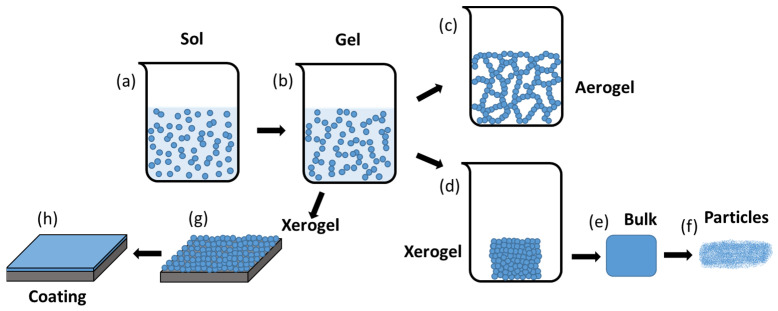
Sol-gel synthesis process (sol (**a**) to gel (**b**)) and generation of ceramic materials (aerogel (**c**), and xerogel (**d**,**g**)) with different morphologies (coating (**h**), bulk (**e**), and particles (**f**)). Adapted from C.J. Brinker et al. [15].

Sol-gel is a physical-chemical technology that allows obtaining dense and homogeneous vitreous and ceramic materials at low temperatures. The synthesis process can be divided into different stages shown in Figure 2. In the first stage (Figure 2a), hydrolysis (Equation (1)) and condensation (Equation (2)) reactions take place in a colloidal solution composed by the mixing of the precursor compounds, catalyst, water, and, in some cases, a co-dissolvent. The most used precursors in the sol-gel process are metal alkoxides because they react readily with water, represented as (M(OR)n) in Equation (1), where M is the metallic element and R can represent a proton or other ligands such as an alkyl or alkoxy groups.

(1) $$M{\left(\mathrm{OR}\right)}_{n}+xH_{2}O\;\to {\left(\mathrm{OH}\right)}_{x}-M{\left(\mathrm{OR}\right)}_{n-x}+x\mathrm{ROH}$$

(2) $${\left(\mathrm{OR}\right)}_{n-x}M-{\left(\mathrm{OH}\right)}_{x}+{\left(\mathrm{OH}\right)}_{x}-M{\left(\mathrm{OR}\right)}_{n-x}\;\to {\left(\mathrm{OR}\right)}_{n-x}M-O-M{\left(\mathrm{OR}\right)}_{n-x}+H_{2}O$$

Once the hydrolysis is initiated, a condensation reaction takes place between partially hydrolyzed molecules, leading to the generation of larger molecules due to a polymerization process. The random interconnections of the larger molecules end up with the formation of a three-dimensional structure. At this point, the second stage of the sol-gel process starts with the formation of the gel (Figure 2b). A gel is a stable porous solid immersed in a liquid medium, which fills the pores inside the three-dimensional structure. The sol to gel transition (gelation) leads to an exponential increase in the viscosity value of the solution.

Once the gel is generated, in the third stage it is necessary to remove the liquid phases present in the gel to obtain a solid material. Thus, a drying process is applied and depending on this process two different materials can be obtained. If the liquid phase present in the gel is removed by a supercritical drying process, a solid material called aerogel is obtained (Figure 2c). This material is mostly air with a solid volume fraction of around 1% [15]. On the other hand, if the liquid phase present in the gel is dried by a slow evaporation process under normal conditions, a fragile solid called xerogel is obtained (Figure 2d). In the final stage of the synthesis process, a sintering treatment is necessary to obtain a dense, pore-free, and homogeneous material from the xerogel (Figure 2e). That would be the normal procedure to obtain a bulk material that can be milled to obtain sol-gel particles for different applications (Figure 2f). However, if a coating is intended to be generated, an intermediate step is necessary to deposit the sol-gel material on the surface of the substrate meant to be coated. The gel is applied on the surface of the substrate and then drying takes place, obtaining a xerogel coating (Figure 2g). Several methods exist to deposit the sol-gel material on the substrates to create a coating. The most extended method is dip-coating [16,17], which consists of the immersion of the substrate in the sol-gel and then the withdrawal of the substrate at a controlled speed to create a uniform coating on the surface of the substrate. This method is widely used due to its simplicity, low cost, and the possibility of coating complex morphologies, but other coating methods such as spin- or spray-coating have been described in the bibliography [18,19,20,21]. However, to generate a dense and homogeneous ceramic layer, a sintering process must be carried out (Figure 2h). Through these stages, vitreous and ceramic coatings and particles can be generated by the sol-gel process.

**Figure 3:**
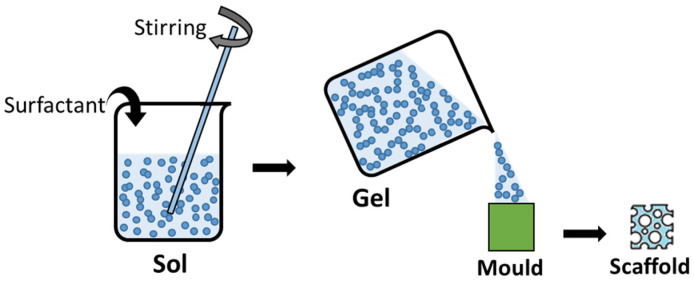
Generation of scaffolds for tissue engineering applications from the sol-gel synthesis process.

Moreover, other morphologies can be generated by modifications in the main sol-gel synthesis stages. For example, sol-gel foam scaffolds can be obtained by the addition of a surfactant and the application of vigorous agitation to the initial sol which, before the total gelation, is cast in a mold with the final porous scaffold morphology [22,23]. After the drying of the gel, a solid scaffold is obtained (Figure 3).

**Figure 4:**
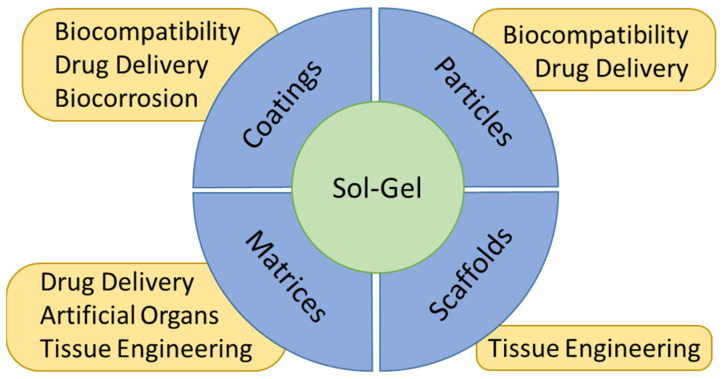
Different uses of sol-gel materials applied in the biomedical field.

The versatility of the sol-gel process allows for its use for the generation of biomaterials with different morphologies and purposes, as shown in Figure 4.

The mechanical stability and integrity of the different sol-gel structures and materials is an important parameter that determines their performance. For example, if a sol-gel coating is generated for biocorrosion protection or biocompatibility improvement of the coated substrate, it is of great importance to avoid the generation of cracks that could decrease the protective behavior or could negatively affect the biocompatibility of the coating itself. It is known that the critical thickness of the sol-gel coatings above which cracks are generated depends on different factors and the precursors used in the generation of the sol-gel [24].

Sol-gel materials, xerogels, and aerogels are brittle, and this behavior influences their performance when used for the generation of coatings, scaffolds, or matrices. However, there are several strategies to decrease the brittleness of these materials including, for example, regulating the precursors used in the sol-gel synthesis or introducing polymer reinforcements in the sol-gel structures. [25,26].

In the case of sol-gel particles used as drug delivery systems, the mechanical stability of the particles influences the liberation rate. Therefore, it is important to control it to obtain a suitable drug release profile. If not, higher or lower delivery rates could lead to the failure of the treatment [12].

## 2. Sol-Gel Coatings for Biomedical Applications

### 2.1. General Considerations

**Figure 5:**
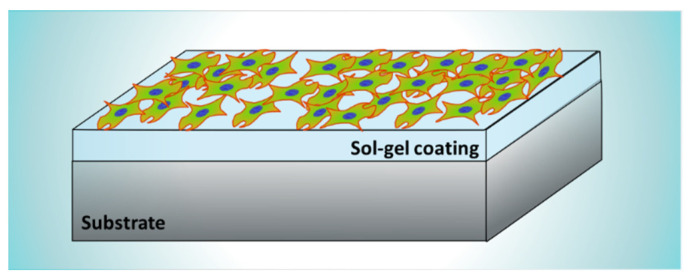
Schematics of cell culture proliferation on a sol-gel coating deposited on a metallic substrate.

The materials synthesized by the sol-gel method are widely used to generate multifunctional coatings that can improve or add new properties to the coated substrates. In biomaterials, the main property that coatings can provide is the improvement of the biocompatibility of the implants. For some materials, that means an improvement in the bioactivity of the implanted materials, enhancing the interaction and the bonding between the body tissues and the implant, by an increment of the cellular growth on their surface, and at the same time decreasing the rejection rate (Figure 5).

Moreover, the possibility of antibacterial properties could help to avoid the bacterial colonization of the implants and, therefore, the risk of infection and rejection of the implanted material. This could be the case for some commercial steel and titanium alloys [19,27,28,29,30,31,32,33].

**Figure 6:**
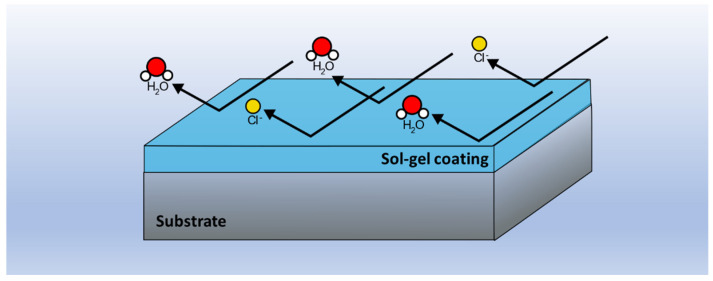
Schematics of a sol-gel coating preventing the corrosion of a metallic substrate by acting as a physical barrier against aggressive elements.

In other cases, when the implants are made from materials that are not only biocompatible but bioactive, as in the case of magnesium alloys, the coatings generated by sol-gel can be used to overcome the drawbacks of these materials. In the case of magnesium alloys, their main challenge is their high reactivity [34]. However, when magnesium implants are coated with a sol-gel coating, the substrates can be isolated from the aggressive medium, decreasing their degradation rate and making them optimal for biological applications (Figure 6).

**Figure 7:**
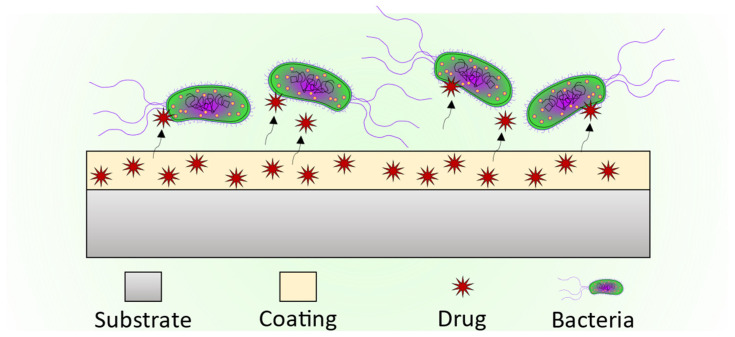
Schematics of a sol-gel coating loaded with an antibiotic drug.

The versatility of the sol-gel synthesis allows adding different particles and substances during the generation of the coating to create functionalized sol-gel materials with improved biocompatibility and antimicrobial or biocorrosion protection properties (Figure 7).

Different materials obtained by the sol-gel process and used to generate coatings can be found in the literature. Among the most common, hydroxyapatite (Ca_5_(PO_4_)_3_(OH)) and bioactive glass systems (BG, SiO_2_-CaO-P_2_O_5_) and different oxides (SiO_2_, TiO_2_, ZrO_2_) are used to improve the biocompatibility and to provide bioactive properties to metallic substrates [35,36,37,38,39].

### 2.2. Coatings for Biocompatibility Improvement

To improve the biocompatibility of titanium and titanium alloys (grade II, grade IV, and Ti6Al4V), surgical stainless steel (316L SS), and other materials which are commonly used for the manufacture of implants (CoCrMo alloys, NiTi alloy), the use of Hydroxyapatite (HAp) coatings is proposed in several scientific papers to improve the osseointegration of these materials [27,28,40]. HAp is a bioactive material, made out of calcium and phosphorous, which promotes the colonization and proliferation of bone cells [41]. Thus, a HAp coating can act as an interface between the metallic substrate and the bone tissue, improving the osseointegration of the implant. To generate HAp by the sol-gel method, different Calcium and Phosphorus precursors are used. Some of the most common are Calcium Nitrate Tetrahydrate (Ca(NO_3_)_2_-4H_2_O), Triethyl Phosphate (C_6_H_15_O_4_P), Phosphorus Pentoxide (P_2_O_5_), Phosphoric Acid (H_3_PO_4_), and Calcium Ethoxide (C_4_H_10_CaO_2_) [5,19,35,40,42]. 

**Figure 8:**
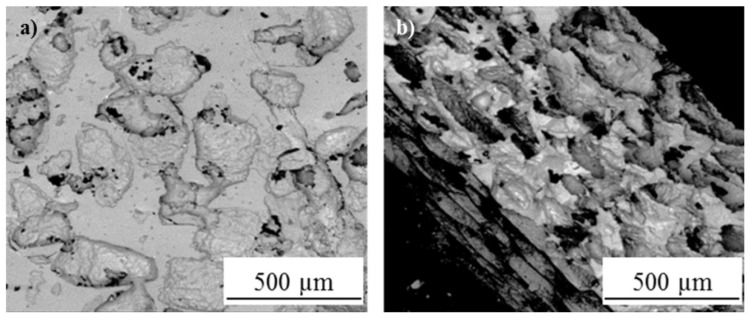
Adapted with permission from C. Domínguez-Trujillo et al. [28]. Copyright 2018 Elsevier. License number 5318790646724. SEM micrographs of the HAp coatings on porous titanium substrates. (**a**) Plain view, (**b**) cross-section.

D.M. Liu et al. [27] generated dense and firmly attached thin hydroxyapatite films on 316L stainless steel substrates. Similarly, A. Balamurugan et al. [35] obtained dense HAp coatings on 316L stainless steel substrates and demonstrated their protective behavior against corrosion processes when immersed in a simulated body fluid environment. C. Domínguez-Trujillo et al. [28] used porous (grade IV) titanium substrates, over which HAp coatings were generated (Figure 8).

**Figure 9:**
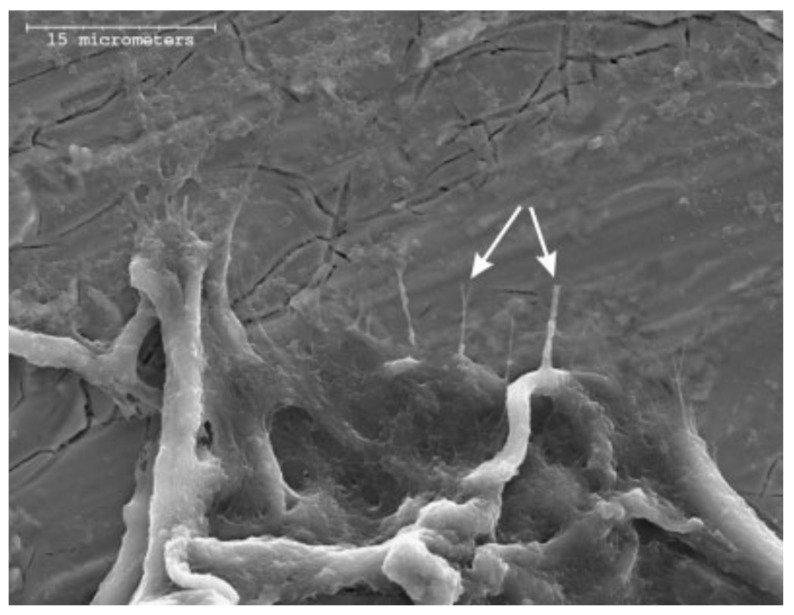
Adapted with permission from S. Areva et al. [50]. Copyright 2004 Elsevier. License number 5318860867897. Collagen fibers attach directly to a titania coating from mature fibroblasts.

Porosity by itself is an important parameter that influences the cellular colonization and growth on the surface of an implanted material. The biocompatibility and osseointegration can be further enhanced if the porous structure is coated with a HAp coating. Thus, the combination of the sol-gel/dip-coating processes used in this research allowed the infiltration of the HAp inside the interconnected pores present in the substrate. Due to its nature, the sol-gel synthesis method combined with one of the different coating methods can be used to generate multilayered or composite coating systems. The generation of composite coatings made out of HAp combined with some titanium or silicon oxides is commonly found in scientific literature. For example, P.A. Ramires et al. [19] used HAp/TiO_2_ and bioactive glass (BG)/TiO_2_ composite coatings to improve the biocompatibility and osseointegration of pure (grade II) titanium dental implants (Figure 9). They developed in vitro and in vivo tests and found that dental implants coated with the HAp/TiO_2_ and BG/TiO_2_ composites presented better performance compared with the uncoated titanium substrates. The presence of HAp stimulated the response of the osteoblastic cells which are in charge of the generation of bone tissue. Moreover, the presence of HAp led to an increase in the bone–implant contact area.

Another example of the use of HAp/TiO_2_ composite coatings was described by D. Sidane et al. [5]. They coated 316L SS substrates with a monolayer of HAp/TiO_2_ composite coatings and with a bilayer of TiO_2_ (inner layer) and HAp (outer layer) coating system. The evaluation of the corrosion protection behavior of the different systems and their biocompatibility by stem cell proliferation tests showed good results in terms of corrosion protection and cell proliferation and differentiation with the use of the HAp/TiO_2_ composite coating.

Despite HAp and calcium phosphate systems being the most used biomaterials to generate coatings to improve the biocompatibility, especially important in the case of bone implants and dental or maxillofacial surgeries due to their outstanding osseointegration, other materials such as Silica (SiO_2_), Titania (TiO_2_), and Zirconia (ZrO_2_) are commonly used for the generation of coatings to improve the biocompatible properties of bioinert materials. In scientific literature, several examples can be found about the synthesis process and the most commonly used precursors to generate these materials. In the case of SiO_2_ coatings, several silicon alkoxides are used as precursors. Three of the most common are Tetraethoxysilane (Si(OC_2_H_5_)_4_), Tetramethoxysilane (Si(OCH_3_)_4_), and Methyl-triethoxysilane (CH_3_-Si(OC_2_H_5_)_3_) [39,43,44,45]. For the TiO_2_ coatings, the use of Tetrabutyl Orthotitanate (Ti(OC_4_H_9_)_4_), Tetraisopropyl Orthotitanate (Ti((CH_3_)_2_CHO)_4_), and Titanium isopropoxide (Ti(OCH(CH_3_)_2_)_4_) as precursors is described in the bibliography [5,46,47]. The use of ZnO and ZrO_2_ as base materials for the generation of coatings through the sol-gel method was also found in the scientific literature [48,49]. For these materials, Zirconium propoxide (Zr(OC_3_H_7_)_4_) and Zinc acetate dehydrate (Zn(CH_3_COO)_2_-2H_2_O) are usually used as source precursors for Zr and Zn.

**Figure 10:**
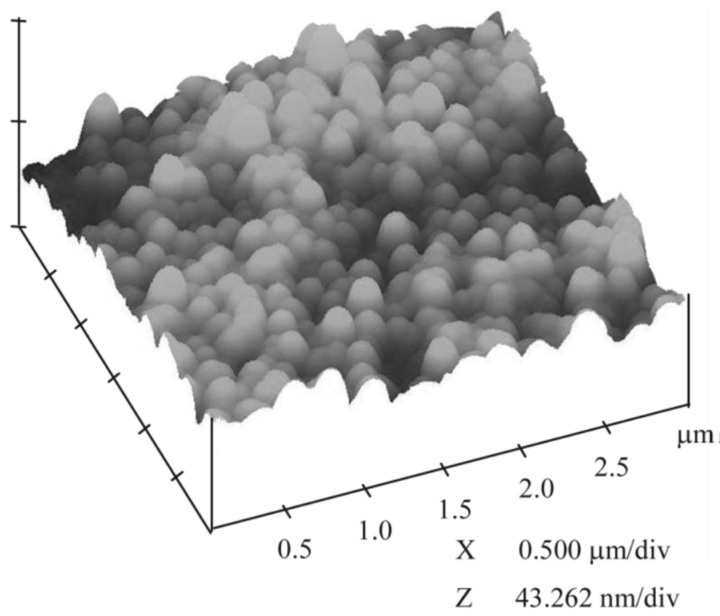
Adapted with permission from Jing-Xiao Liu et al. [51]. Copyright 2003 Elsevier. License number 5318830382484. AFM image of TiO_2_ coating generated by sol-gel and deposited on NiTi alloy.

In their research, Sami Areva et al. generated nanoporous Titania coatings deposited by dip-coating on Grade II titanium substrates, using Tetraisopropyl Orthotitanate (Ti((CH_3_)_2_CHO)_4_) as the sol-gel precursor [50]. They developed biocompatibility in in vitro and in vivo experiments, using simulated body fluid (SBF) mixed with three different protein solutions (fibrinogen, bovine serum albumin, and plasma solution) and Long-Evans rats as an animal model, respectively. The assessment of the surface of the Titania coatings after immersion over 14 days at 37 °C in the different solutions revealed that Ca/P phases were formed only on the coatings immersed in pure SBF, while the presence of proteins in the other SBF solutions inhibited the growth of the initially formed Ca/P. From in vivo tests, they achieved excellent bonding between the soft tissues and the sol-gel-derived Titania coated implants, while non-coated titanium implants showed no evidence of soft tissue attachment. A mild inflammatory response was found for both titania-coated and non-coated grade II titanium substrates. Another example of the use of titania coatings generated by sol-gel to improve the biocompatibility of metallic substrates was reported by Jing-Xiao Liu et al. They generated TiO_2_ coatings using Tetrabutyl Orthotitanate (Ti(OC_4_H_9_)_4_) as the precursor, and dip-coating as deposition technique on NiTi surgical alloy (Figure 10). They carried out corrosion and biocompatibility in vitro experiments using Tyrode’s solution and human blood as test media respectively, finding that the presence of TiO_2_ coatings improved the behavior against corrosion of the composite material, increasing the breakdown potential by +200 mV and decreasing the current density in one order of magnitude compared with the values obtained for the bare NiTi substrate. Moreover, the TiO_2_-coated samples showed longer clotting times and a lower number of adhered platelets after 1 h of immersion in human blood, indicating that TiO_2_-coated samples show better blood biocompatibility than the bare NiTi substrates [51]. Thus, the use of Titania coatings can improve the performance of cardiovascular stents made from NiTi alloy.

One example of the generation of silica coatings was described by M. Gerritsen et al. In their research, they evaluated the biocompatibility of sol-gel coatings meant to be used to optimize the properties of implantable glucose sensors [52]. They developed SiO_2_ coatings using Tetraethoxysilane (Si(OC_2_H_5_)_4_) as the precursor for the sol-gel synthesis and deposited them by spin-coating onto Polyethylene Terephthalate and Polystyrene substrates. Moreover, the coatings were doped with different organic macromolecules such as heparin, dextran sulphate, Nafion^®^, polyethylene glycol, and polystyrene sulphonate. They assessed the soft tissue biocompatibility of the doped SiO_2_ coatings using MEM α-medium and human dermal fibroblasts for the in vitro tests and rabbits as an animal model for the in vivo tests. They found out that although these coatings presented biocompatibility, their biocompatibility was lower than that of bio-glasses or titanium implants. Another example of the use of SiO_2_ coatings to improve the biocompatibility of metallic substrates was reported by A. Durán et al. [30]. They used Tetraethoxysilane (Si(OC_2_H_5_)_4_) and Methyl-triethoxysilane (CH_3_-Si(OC_2_H_5_)_3_) as precursors to generate SiO_2_ coatings deposited by dip-coating on 316L stainless steel, Ti6Al4V, and CrCoMo alloy substrates. Moreover, the SiO_2_ coatings were doped with SiO_2_·CaO·P_2_O_5_ (Bioactive glass) particles. They carried out in vitro and in vivo tests using simulated body fluid (SBF) and Hokkaido rats as an animal model, respectively. They found that the presence of the coatings always improved the corrosion behavior and the formation of hydroxyapatite deposits on all the coated metallic substrates. In vivo tests showed no rejection or inflammatory reactions. Moreover, the formation of bone tissue and vascular development was observed for endomedullar CrCoMo alloy implants coated with a combination of SiO_2_ and SiO_2_·CaO·P_2_O_5_ coatings.

### 2.3. Coatings for Biocorrosion Protection

There are some materials whose main drawback is not biocompatibility but their biodegradation rate in physiological conditions. That is the case of some magnesium alloys such as AZ31B and AZ91D or other Al-free magnesium alloys such as Mg-Zn-Ca and Mg-Zn-Zr alloys. Magnesium and magnesium alloys are interesting for biomedical applications due to their mechanical properties. Magnesium is the lightest of the structural metals used in biomedicine, with density and stiffness values close to that of natural bone tissue. Thus, the use of magnesium implants helps to avoid the probability of implant failure due to the stress-shielding effect [53,54]. This phenomenon is caused by the presence of a metallic implant with a value of elastic modulus much higher than the bone tissue that surrounds it. In this case, the load that should affect the bone tissue is borne by the metallic implant, which promotes osteopenia due to the resorption of the unloaded bone, as described by the concept of bone functional adaptation to mechanical loadings [55,56,57]. Moreover, magnesium is a biocompatible and bioresorbable material and Mg^2+^ cations are present in different biochemical and physiological processes. However, magnesium presents high reactivity and corrosion rates [58,59,60,61,62,63]. To overcome this problem, the use of the sol-gel synthesis method to generate SiO_2_, TiO_2_, and other kinds of inorganic coatings on magnesium alloy substrates is proposed by several authors to protect them from the aggressive media and to control their degradation rate in biological environments and therefore improve their biocompatibility. For example, Y. Castro et al. [34] generated SiO_2_ coatings with and without the addition of colloidal silica particles. The coatings were deposited by dip-coating on AZ31B and AZ91D magnesium substrates to control their degradation rate. After 28 days of immersion in simulated body fluid solution, hydrogen evolution tests combined with pH measurements and potentiodynamic tests were carried out to assess the corrosion behavior of the substrates treated with the different coating conditions. They found differences in the corrosion behavior depending on the substrate and the composition of the coatings but, in all cases, the use of coatings improved the corrosion behavior compared with the bare substrates. In their research, P. Amaravathy et al. developed niobium oxide (Nb_2_O_5_) coatings by the sol-gel synthesis method to improve the biocompatibility and corrosion protection of AZ31 magnesium alloy substrates. They developed hydrogen evolution and potentiodynamic tests to evaluate the corrosion behavior of the different samples, finding that the presence of the Nb_2_O_5_ coatings considerably decreased the corrosion and degradation rate of the AZ31 substrates [64]. Junhua Hu et al. used TiO_2_ coatings to improve the degradation rate of AZ31 magnesium alloy substrates. They assessed the corrosion behavior of the coated samples immersed in Hank’s solution by gravimetric and electrochemical measurements, obtaining a significant improvement in the corrosion behavior of the coated samples with a dependence on the annealing temperature of the coatings [65].

### 2.4. Coatings as Drug Delivery Systems

**Figure 11:**
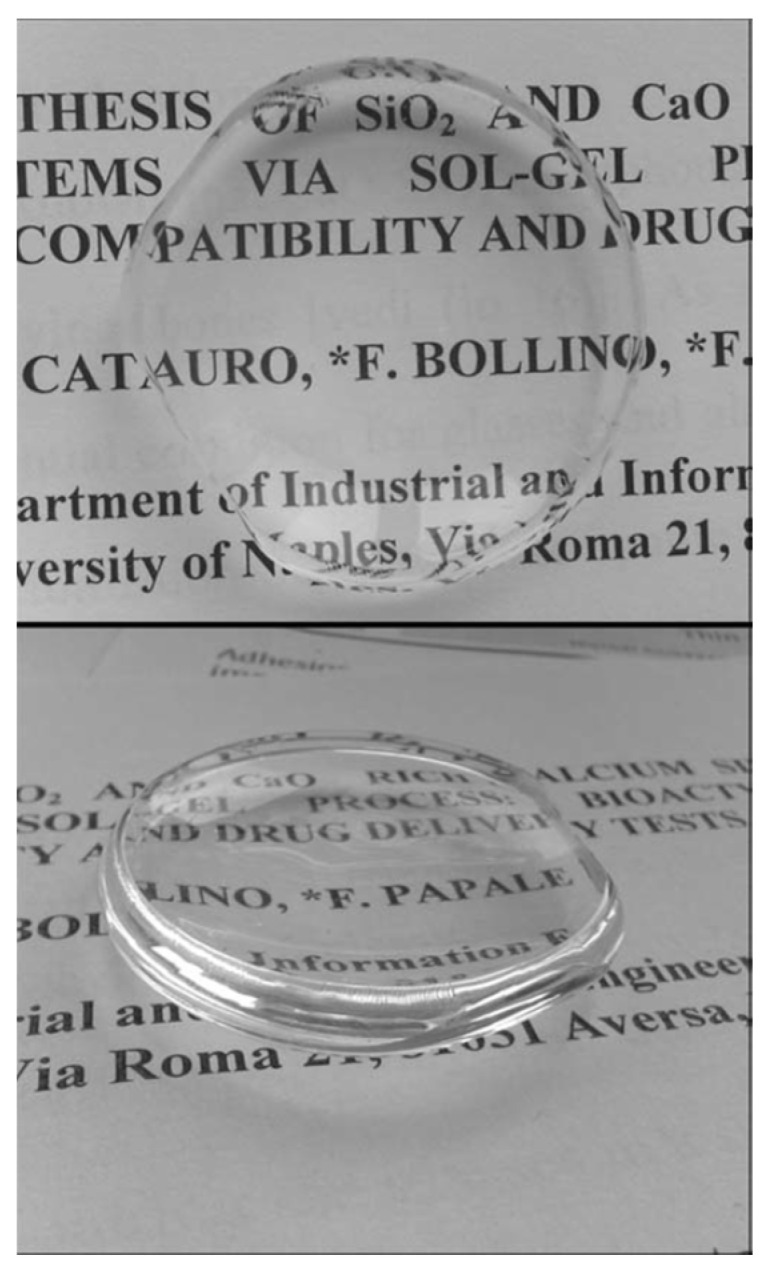
Adapted with permission from M. Catauro et al. [66]. Copyright 2013 Elsevier. License number 5318840223264. Ca/Si ampicillin doped bulks.

One important advantage of the sol-gel process is that it allows the addition of several particles and substances during the synthesis that can provide specific properties to the final coatings and particles system. For example, these coatings could be loaded with a wide range of drugs to serve not only as bioactive and protective barriers but accurate drug delivery systems that could release the loaded drug in the specific place where the coated material is implanted. In their research, M. Catauro et al. loaded Silica (SiO_2_) and Calcium Silicate (Ca/Si) amorphous materials with sodium ampicillin, a broad-spectrum antibiotic (Figure 11), and studied the release kinetics of the antibiotic from the materials [66]. To improve the antibacterial behavior of the coatings, the addition of silver (Ag) particles and compounds during the sol-gel synthesis process is described in the scientific literature by several authors [42,43,49]. Silver ions (Ag^+^) have demonstrated a strong antibiotic effect [67,68,69].

**Figure 12:**
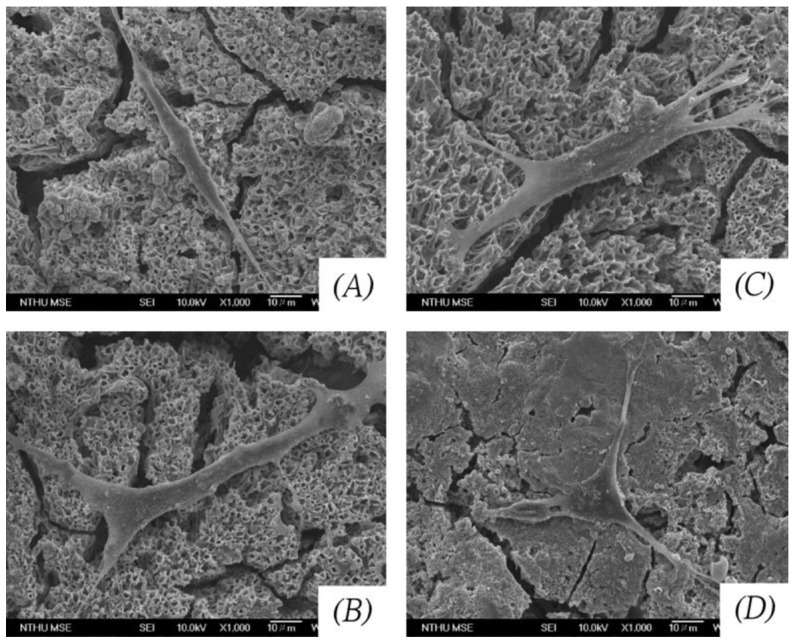
Adapted with permission from Chung et al. [42]. Copyright 2005 Elsevier. License number 5318840713059. SEM images of HGF-1 cells attached on HAp coatings. (**A**) Pure HAp, (**B**) HAp + 1000 ppm Ag, (**C**) HAp + 1000 ppm Zn, (**D**) HAp + 10,000 ppm Zn.

Chung et al. developed hydroxyapatite coatings loaded with Ag^+^ ions (Figure 12) and assessed the in vitro antimicrobial effect of these coatings against the Streptococcus mutans (S. *mutans*) bacterial strain, avoiding the bacterial growth on the HAp coating. They also loaded the HAp coatings with Zn^2+^ ions, which also provided antimicrobial properties to the coatings. Moreover, the biocompatibility of these coatings was evaluated using human gingival fibroblasts (HGF-1) [42]. Z.N. Kayani et al. generated Ag-doped ZnO thin films with different Ag contents and studied the in vitro antimicrobial effect on Staphylococcus aureus (S. *aureus*) and Pseudomonas aeruginosa (P. *aeruginosa*) cell strains, obtaining the same antibacterial effect for all the coatings, regardless of the Ag concentration [49].

Not only drugs can be loaded into sol-gel coatings. A. Durán et al. used bioactive glass particles belonging to the CaO-SiO_2_-P_2_O_5_ system to load hybrid Tetraethoxysilane and Methyl-triethoxysilane coatings [30]. Using the loaded coatings, they achieved an improvement against corrosion processes. Moreover, in vitro and in vivo tests were developed in this research. Their results showed that in vitro biocompatibility was achieved due to the formation of HAp deposits after 7 days of immersion in simulated body fluid (SBF) and Hank’s balanced salt solution (HBSS). In vivo tests showed no rejection of the implanted materials treated with the loaded hybrid coatings and the formation of new bone tissue. 

## 3. Sol-Gel Particles for Biomedical Applications

**Figure 13:**
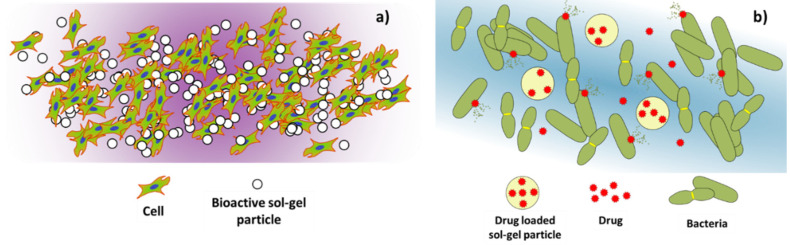
(**a**) Schematics of bioactive loaded sol-gel particles promoting cell proliferation and differentiation, (**b**) schematics of sol-gel particles releasing antibiotic drugs for hindering bacteria proliferation.

As previously described in the sol-gel synthesis section, in addition to coatings, powder materials can be generated from the milling of the bulk materials which are obtained after the sintering process of a xerogel. Other methods such as spray drying can be used to generate sol-gel particles. Spray drying is a technique where dry powders composed of micro- and nanoparticles can be obtained from a liquid solution (sol-gel) [70,71,72]. Thus, macroscopic as well as nanoscale particles can be obtained and used for several biomedical purposes such as biocompatibility improvement of biomaterials, restoration of damaged tissues, and drug delivery systems. Additionally, the sol-gel synthesis method to obtain bioactive particles is interesting for stem cell therapies. For example, the use of Zn or Sr loaded sol-gel bioactive glass granules was demonstrated to be an effective way to improve the growth and the osteogenic differentiation of mesenchymal stem cells [73,74] (Figure 13).

The sol-gel micro- and nanoparticles can be loaded with different substances such as drugs, growth factors, proteins, etc., that can influence the growth and differentiation of cell cultures, and the prevention of bacterial infections due to the presence of these particles. As the loaded substances are liberated to the extracellular medium from the particles, the cells can assimilate them to differentiate into specific tissue, for example, promoting the osteogenic or angiogenic differentiation to generate bone or vascular tissues [75]. On the other hand, specific drugs delivered from the sol-gel particles can prevent bacteria proliferation by attacking and destroying the bacterial cell wall, killing the bacteria, hindering the bacterial adhesion to living tissues, or interfering with the bacterial growth mechanisms at a molecular scale, preventing the spreading of bacterial infections [76,77].

### 3.1. Particles for Biocompatibility Improvement

**Figure 14:**
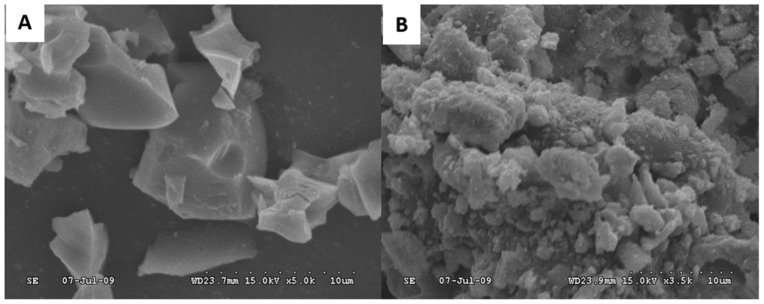
Adapted with permission from W. C. Liu et al. [81]. Copyright 2019 Elsevier. License number 5318870269625. SEM images of (**A**) C_3_S and (**B**) Hap/C_3_S (C-50) particles.

In the case of biocompatibility improvement, bioactive glass particles can be generated by sol-gel and then added to different materials to increment their biocompatibility due to the demonstrated effect of bioactive glasses to induce bone cell growth and osseointegration when they are put in contact with natural bone tissue [78]. One example of the use of bioactive particles generated by sol-gel and used to improve the biocompatibility of other materials was exposed by G.M. Luz et al. [79]. In their research, they developed two different bioactive glass nanoparticles (Figure 14), SiO_2_-CaO-P_2_O_5_ and SiO_2_-CaO systems, as well as SiO_2_-CaO-P_2_O_5_/Chitosan composites. The bioactivity of the different nanoparticles was assessed in vitro by immersion of the nanoparticles in simulated body fluid (SBF), finding their ability to induce mineralization, which means that these nanoparticles present desired characteristics of biomaterials used in bone tissue engineering. The non-toxicity of the nanoparticles was evaluated by contact of the nanoparticles with L929 mouse fibroblasts cultures. The SiO_2_-CaO-P_2_O_5_/Chitosan composites were found to be osteoconductive with potential uses in tissue regeneration treatments.

The use of different silicon-based materials, such as tricalcium silicate or glass ionomer cement, which are also known as bone cement, is widespread in orthopedics and dentistry to fill gaps and defects in fractured bones or tissues affected by tumors. In this area, different research can be found in the scientific literature where calcium phosphate particles are generated by sol-gel and added to the bone cement to increase their biocompatibility and mechanical properties. In their research, A. Moshaverinia et al. studied the effect of the addition of hydroxyapatite and fluoroapatite nanobioceramics, generated by sol-gel, into a commercial glass ionomer cement. They found that the addition of the sol-gel generated nanohydroxyapatite and nanofluoroapatite powders to the commercial glass ionomer cement enhanced their mechanical properties. Moreover, the bonding strength of the doped cement with bone tissue was increased compared with the commercial cement [80]. Another example of the use of the sol-gel method to generate hydroxyapatite and tricalcium silicate powders for the development of bone cement was described by W.C. Liu et al. [81]. In this research, tricalcium silicate powders were synthesized using tetraethyl orthosilicate and calcium nitrate as the precursor in the sol-gel process, and then hydroxyapatite sol was generated using triethyl phosphate and calcium nitrate tetrahydrate as precursors. The previously obtained tricalcium silicate powders were added to the hydroxyapatite solution to finally obtain a mixture of hydroxyapatite/tricalcium silicate composite powders. In vitro biocompatibility tests of the composite powders were developed using L929 and MG-63 cell lines.

They found that the presence of hydroxyapatite improved the biocompatibility of the tricalcium phosphate cement by a reduction of the pH of the medium. However, different hydroxyapatite/tricalcium phosphate proportions were studied and some of them led to a decrease in the mechanical properties. The composite with 25% hydroxyapatite/75% tricalcium phosphate composition showed good biocompatibility and relatively high compressive strength, being interesting for the development of bone cement. Bone cement can also be used for dental applications. V.V. Anusha Thampi et al. used the sol-gel method to generate hydroxyapatite and nanobioactive glass powders for a dental cement nanocomposite by mixing the generated hydroxyapatite and nanobioactive glass with alumina/zirconia nanocomposite, previously prepared through spray pyrolysis technique. The hydroxyapatite powders were prepared using Ca(NO_3_)_2_-4 H_2_O and NH_4_H_2_PO_4_ as precursors. In the case of the nanobioactive glass particles, Tetraethyl Orthosilicate, Triethyl Phosphate, and Calcium Nitrate Tetrahydrate were used as precursors in the sol-gel process. The different compounds were mixed in a powder state to obtain the new cement composite (HANBG) and then their mechanical, as well as bioactivity properties were compared with that of a glass ionomer cement (GIC) and a glass ionomer cement and alumina/zirconia composite (GICAZ). They found that the new HANBG cement composite showed higher hardness and young’s modulus values than commercial glass ionomer cement and the GICAZ. Moreover, HANBG cement showed bioactivity and antimicrobial activity. Thus, the sol-gel method can be used to generate bone cement for dental applications [82].

### 3.2. Particles as Drug Delivery Systems

One of the most important features of the nanoparticles generated by sol-gel is the possibility to load them with different molecules to be used as drug delivery systems. Moreover, the possibility to locate the drug-loaded nanoparticles with precision in the specific place where an organ is damaged or the treatment is needed makes them an accurate tool for minimally invasive treatments.

In their research, P. Kortesuo et al. [70] generated sol-gel spray-dried silica-gel microspheres using Tetraethyl Orthosilicate as silica precursor in the sol-gel process. The microspheres were loaded with dexmedetomidine (sedative drug) and toremifene (cancer treatment drug), which were directly added to the solution during the sol-gel process. The release rate of the different drugs from the silica microspheres was assessed in vitro, soaking them in simulated body fluid (SBF). They found that the release rate was dependent on the initial drug concentrations in the microspheres.

**Figure 15:**
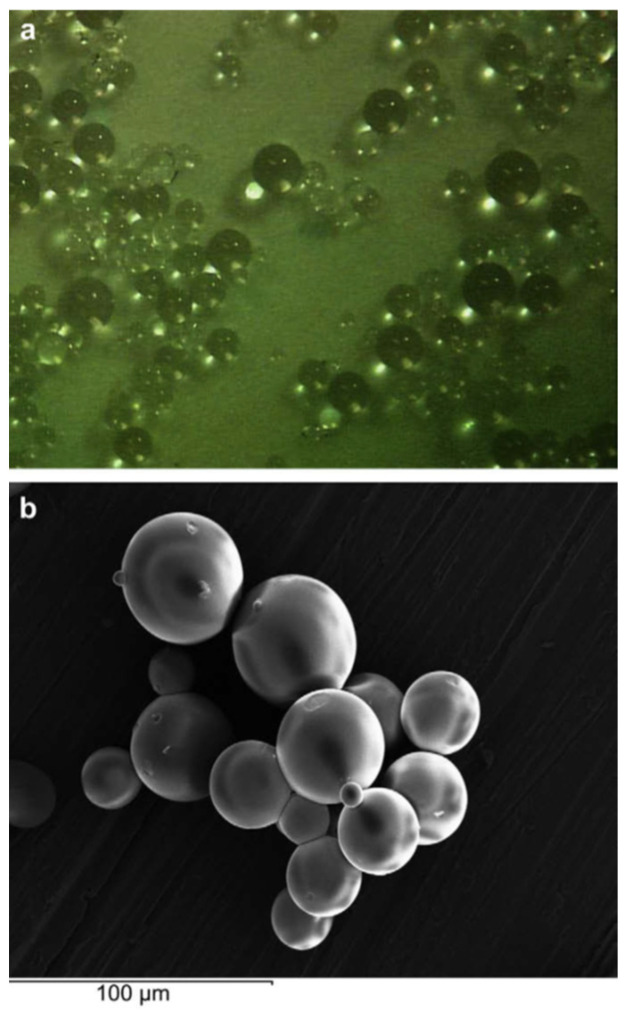
Adapted with permission from S. Radin et al. [83]. Copyright 2009 Elsevier. License number 5318830763811. (**a**) and (**b**) are optical and SEM micrographs of emulsified acid-base catalyzed silica microspheres.

S. Radin et al. [83] generated silica microspheres using Tetraethyl Orthosilicate as the precursor in the sol-gel synthesis process (Figure 15). Moreover, two pharmaceutical molecules, vancomycin (antibiotic) and bupivacaine (analgesic), were incorporated into the sol to obtain loaded silica microspheres intended to be used as drug carriers for drug delivery systems. They used an acid-base catalysed sol-gel process followed by emulsification to control the gelation time and the size of the microspheres. To obtain the silica microspheres, the emulsified sol-gel was added dropwise onto vegetable oil and stirred until the microspheres precipitated. Then, the microspheres were filtered, rinsed with deionized water, and dried. To assess the in vitro release and the degradation rate of the microspheres, they were immersed in phosphate-buffered saline (PBS) solution at 37 °C. The concentration of released drugs was measured every 24 h. Through this acid-base catalysed hydrolysis followed by the emulsification of the synthesized sol, they achieved the generation of control-release silica sol-gel microspheres suitable for drug delivery systems.

Silicon oxide particles are commonly generated by sol-gel for their use as drug delivery systems. However, as in the case of coatings, calcium and phosphorous compounds such as hydroxyapatite show excellent biocompatibility properties and are excellent candidates for the generation of carriers for drug release. In this regard, A.J. Melville et al. generated ibuprofen (anti-inflammatory, analgesic) loaded hydroxyapatite powders and studied the effect of calcination temperature of hydroxyapatite on its drug delivery behavior. The release of ibuprofen was studied by immersion of the loaded hydroxyapatite particles in pH 7.3 and 0.1 M tris buffer solution. The concentration of the released drug was assessed at different immersion times using a UV-visible spectrophotometer. They found that the drug release gradient was increased with increasing calcination temperatures. Moreover, for a stable calcination temperature, the drug release gradient was increased with increasing calcination time. Therefore, they demonstrated that the drug delivery behavior of hydroxyapatite could be changed by the application of different thermal treatments [84].

Ag and Zn ions are usually loaded into coatings and particles to provide them with antimicrobial properties. In this regard, Chung et al. followed the sol-gel route to generate hydroxyapatite particles using calcium nitrate tetrahydrate (Ca(NO_3_)_2_-4H_2_O) and Triethyl phosphate (C_6_H_15_O_4_P) as calcium and phosphorous sources, respectively. Moreover, AgNO_3_ and Zn(NO_3_)_2_-6H_2_O were added during the sol-gel synthesis to obtain Ag- and Zn-loaded hydroxyapatite particles. They assessed the antimicrobial effect of this material using the Streptococcus mutans strain. Ag/Zn loaded hydroxyapatite disks were put in contact with S. mutans cultures in brain heart infusion (BHI) agar plates and Ag/Zn loaded hydroxyapatite particles were immersed in BHI broth. In the case of the agar plates, the antimicrobial effect was achieved, and the growth of the bacterial colonies was inhibited around the Ag/Zn loaded hydroxyapatite disks. However, in the liquid culture medium (BHI broth), no release of Ag and Zn ions from the hydroxyapatite particles was detected and, therefore, no antimicrobial effect was observed [85].

## 4. Sol-Gel Matrices

**Figure 16:**
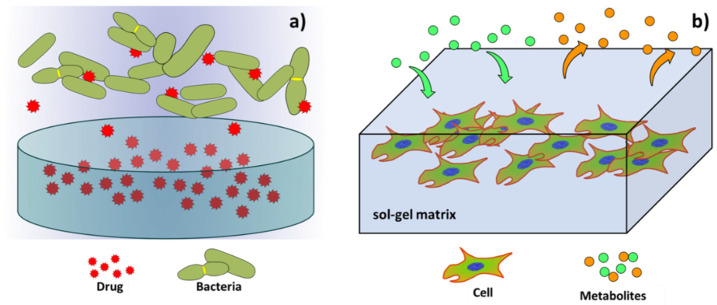
(**a**) Schematics of a sol-gel matrix loaded with antibiotic drugs for the treatment of localized bacterial infections. (**b**) Schematics of a cell culture embedded in a porous sol-gel matrix for tissue engineering applications.

The sol-gel process allows obtaining porous gel matrices, which can be interesting for different uses in the biomedical field, especially for the generation of drug delivery systems (Figure 16a), as well as the generation of bioartificial organs loaded with living cells intended to restore specific cellular processes lost by damaged organs (Figure 16b).

### 4.1. Sol-Gel Matrices for Drug Delivery Systems

As well as in the case of the sol-gel coatings and sol-gel particles, sol-gel porous bulk materials or matrices can be generated, controlling the pore size to be loaded with different molecules or drugs that are intended to be released at a specific rate once implanted in a biological environment, to be used as drug delivery systems. One example of this use of the sol-gel synthesis method was exposed by P. Kortesuo et al. [86]. In their research, they generated silica xerogel discs from tetraethyl orthosilicate and polyethylene glycol precursors and used them as carriers to be used as controlled drug delivery systems. The Xerogel matrix discs were loaded with toremifene citrate, a drug used in breast cancer treatments, and then their dissolution and drug release rates were assessed both in vitro, by immersion in simulated body fluid (SBF), and in vivo, by subcutaneous implantation of the loaded discs in female mice. They found that, despite the silica xerogel being a biocompatible and biodegradable material, the release rate of the drug molecules from the xerogel was difficult to control. Another example of the use of the sol-gel synthesis route to generate porous materials for drug delivery systems was developed by L. Sieminska et al. [10]. In their research, they generated hormone (progesterone, 17β-estradiol, estrone, and hydrocortisone)-loaded porous silica matrices using tetraethyl orthosilicate as the precursor for the sol-gel process. The silica matrices were loaded with hormones using two different methods. In the first method, the hormones were directly added to the sol. The second method consisted of the immersion of the porous silica matrices in hormone solutions. The porous matrices were soaked in the hormone solutions for 5 days and then dried slowly at room temperature. The diffusion of the hormones from the porous silica matrices was assessed by immersion of the loaded silica matrices in different solutions (pure ethanol, 27.5 wt.% ethanol-water, and a physiological solution with pH = 7.4), and the evaluation of the different solutions with UV absorption. They found small diffusion coefficients for the hormones in pores with an average diameter of 3 nm. Thus, this procedure can be used to generate slow drug delivery systems. In their work, M. Hernández-Escolano et al. [87] generated sol-gel matrices using triethoxyvinylsilane and tetraethyl-orthosilicate as precursors. They loaded the sol-gel matrices with procaine, an anaesthetic drug. The release kinetics of the drug from the sol-gel matrices was assessed by immersion of the samples in PBS at 37 °C, and by the use of UV-vis spectrophotometry to determine the concentration of the delivered drugs. The sustained release of the active molecule from the sol-gel matrices over 3 days of immersion was achieved. They found that the degradation of the sol-gel was the main mechanism underlying the control of release.

### 4.2. Sol-Gel Matrices for Tissue Engineering

The sol-gel synthesis process can be used to generate artificial organ research models of interestin tissue engineering. These can be developed due to the versatility of the sol-gel process, which allows the addition of a wide range of particles and substances into the sol-gel during the synthesis process. Thus, instead of drug molecules or nanoparticles, living cells are encapsulated into sol-gel matrices which, under specific conditions and stimuli, can grow and act as small organ models. In this case, the generation of porous sol-gel materials is intended to generate matrices that allow the diffusion of cell nutrients and the elimination of metabolic by-products.

**Figure 17:**
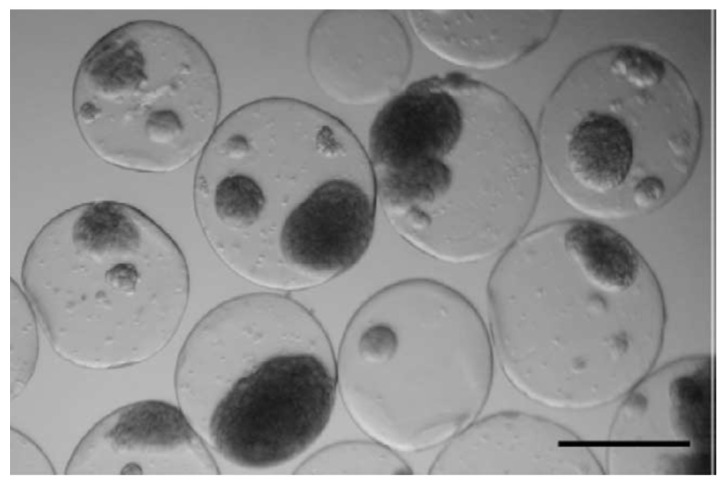
Adapted with permission from S. Sakai et al. [88]. Copyright 2002 Elsevier. License number 5318830910749. Micrograph of encapsulated islets in Alg/AS/Alg microcapsules. The bar represents 500 μm.

In their research on bioartificial organs, Pope, Braun, and Peterson evaluated the behavior of islets of Langerhans, which are the pancreatic regions that contain the endocrine (hormone-producing) cells, encapsulated into silica gel matrices [13]. They controlled the pH, salinity, and temperature conditions during the sol-gel synthesis process to create porous silica gels that were not harmful to live cells, and then loaded them with isolated islets of Langerhans. They carried out in vitro and in vivo tests to assess the insulin secretory response of the pancreatic islets encapsulated into the silica gel, finding no indications of diabetes for non-obese diabetic mice after 3 months of treatment. Another approximation for the creation of a bioartificial pancreas model using the sol-gel synthesis process was exposed by S. Sakai et al. [88]. They developed alginate/aminopropyl-silicate/alginate (Alg/AS/Alg) membranes by a sol-gel process. Then the membranes were loaded with Wistar rat’s pancreatic islets (Figure 17) and transplanted to the peritoneal cavities of male DDY mice with induced diabetes. Transplanted mice developed normoglycemia 24 h after the implantation of the cell-loaded membranes and up to 105 days after implantation, without the administration of immunosuppressive drugs.

**Figure 18:**
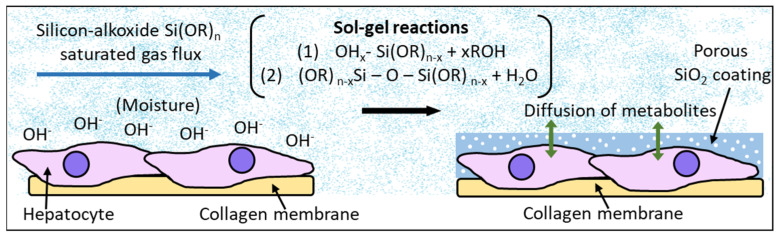
Porous sol-gel silica coatings on cell cultures generated by the biosil method, adapted from the information provided by M. Muraca et al. [90].

In an approach for the future development of a bioartificial liver, M. Muraca et al. assessed the metabolic activity of CD rat hepatocytes encapsulated in combined collagen/silica membranes. Silica membranes, 0.1 µm thick, were generated by the biosil method [14,89] (Figure 18), which is based on the hydrolysis and condensation reactions of a silicon-alkoxide saturated gas phase in contact with the hydroxides present in the moist surface of the cell culture, promoting the sol-gel reaction directly on the cellular surface and generating a silica layer covering the cell cultures. The cell viability of the entrapped hepatocytes was unaffected after the encapsulation process. Moreover, the metabolic activity was evaluated in terms of bilirubin conjugation, ammonia removal, urea synthesis, and diazepam metabolism, resulting as unaffected by the biosil procedure or even enhanced due to favorable changes in the hepatocyte microenvironment [90].

## 5. Sol-Gel Scaffolds for Tissue Engineering

**Figure 19:**
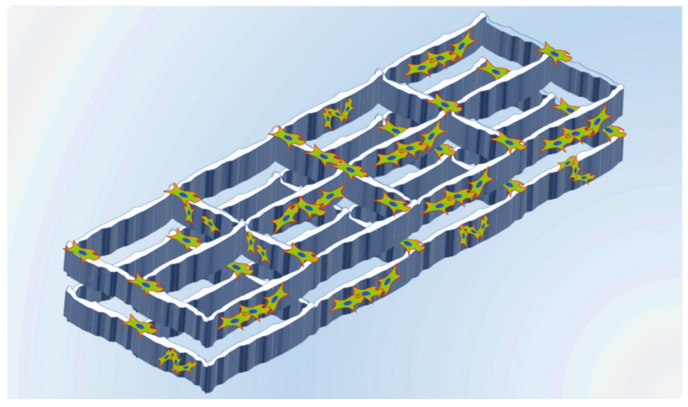
Schematics of a cell culture spreading on the surface of a sol-gel scaffold for tissue engineering applications.

In addition to the generation of artificial organs by the entrapment of living cells inside porous sol-gel matrices, the sol-gel process can be used to generate scaffold structures that are interesting for tissue engineering and wound healing treatments (Figure 19).

**Figure 20:**
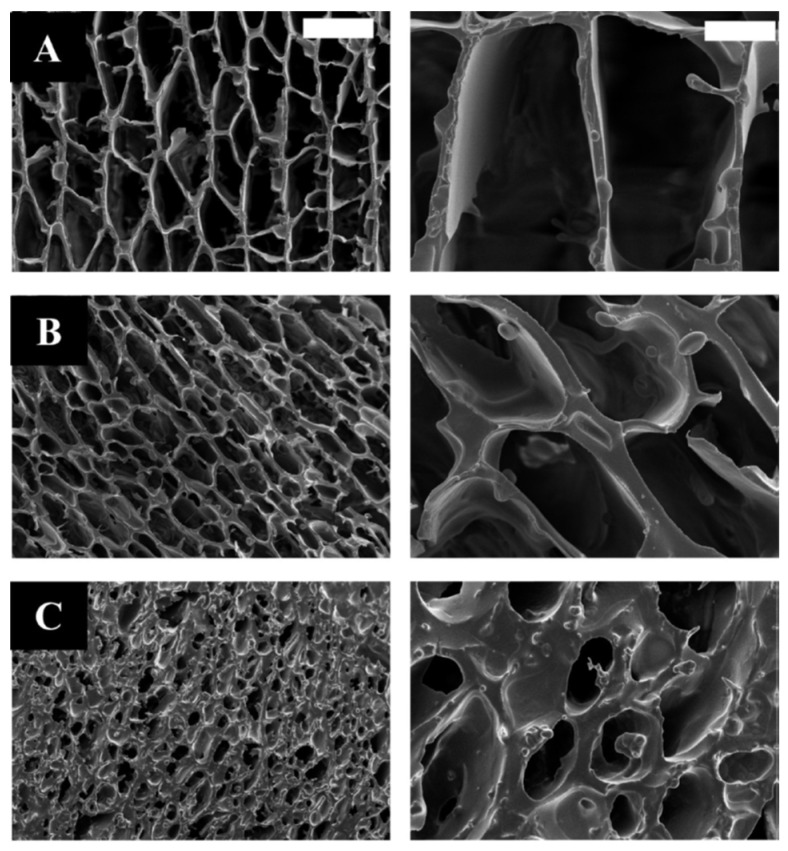
Adapted with permission from Y. Minaberry et al. [92]. Copyright 2011 American Chemical Society. SEM images of cross-sectioned monolithic scaffolds prepared at solid contents and freezing rates of (**A**) 5 wt.%, 1.5·10^−2^ mm s^−1^, (**B**) 7 wt.%, 3·10^−2^ mm s^−1^, and (**C**) 10 wt.%, 6·10^−2^ mm s^−1^. The left scale bar represents 50 μm, the right bar represents 10 μm.

In their research, Qi-Zhi Chen et al. generated foam sol-gel-derived 45S5 Bioglass^®^ scaffolds by the addition of a surfactant and vigorous agitation of the initial sol and then cast them into molds when the gelation of the foamed sol was nearly completed [91]. In vitro tests have shown that these scaffolds supported the proliferation of MG63 osteoblast-like cells. Another method to obtain macroporous bioglass scaffolds generated by the sol-gel method was exposed by Y. Minaberry et al. In their research, a sol was generated from calcium nitrate tetrahydrate (Ca(NO_3_)_2_-4H_2_O), triethyl phosphate, and tetraethyl orthosilicate [92]. After the gelation process, the gel was poured into molds and these molds were unidirectionally frozen into a −196 °C liquid-nitrogen bath. Then the samples were freeze-dried and the obtained green monoliths were finally annealed at 873 K; the procedure described is known as ice-segregation-induced self-assembly (ISISA). Thus, porous monoliths were obtained from a combination of sol-gel and the ISISA method (Figure 20). In vitro bioactivity tests were carried out by immersion of the scaffolds in simulated body fluid (SBF) and incubation at 37 °C for 1 to 3 weeks. After this time, Ca and P deposits were identified on the surface of the scaffolds in a proportion like that of the hydroxyapatite which indicates a well-defined in vitro biomineralization and the potential use of these scaffolds for bone tissue regeneration.

**Figure 21:**
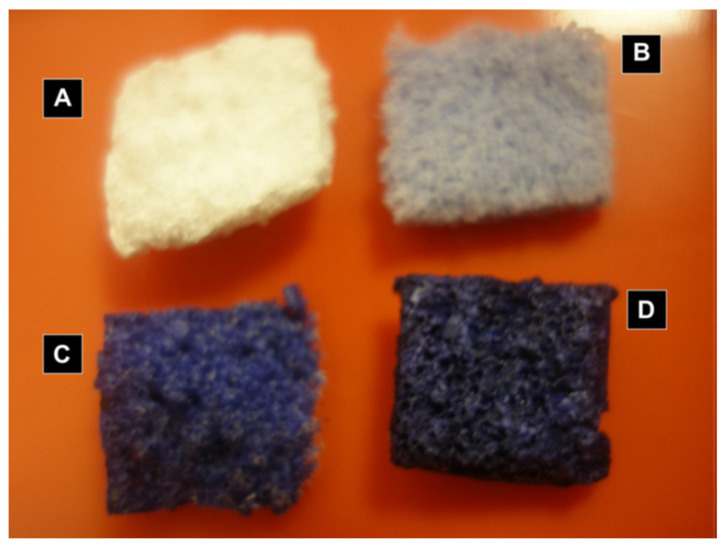
Adapted with permission from M.G. Raucci et al. [93]. Copyright 2010 Elsevier. License number 5318831137579. (**A**) PCL scaffold, (**B**) HAp/PCL scaffold, (**C**) HAp/PCL scaffold after 3 days in SBF solution, and (**D**) HAp/PCL scaffold after 5 days in SBF solution.

M.G. Raucci et al. [93] generated hybrid hydroxyapatite/polycaprolactone (HAp/PCL) scaffolds synthesized by sol-gel in combination with the salt-leaching technique to generate a porous morphology (Figure 21). The sols were generated using calcium nitrate tetrahydrate (Ca(NO_3_)_2_-4H_2_O) and di-phosphorous pentoxide (P_2_O_5_) as hydroxyapatite precursors, and Poly(ε-caprolactone) as the biodegradable polymer. In vitro biocompatibility tests were carried out by immersion of the scaffolds in simulated body fluid (SBF). The presence of hydroxyapatite particles in the composite scaffolds improved the formation of an apatite coating on the surface of the scaffolds during the in vitro tests, which indicates the potential use of these materials for bone-tissue regeneration treatments.

As a final overview of all the different possibilities that the sol-gel process offers to develop materials and structures with interest in the biomedical field, Table 2 summarizes the different sol-gel materials found in scientific literature used in different biomedical applications, depending on their morphology.

## 6. Innovative Applications of the Sol-Gel Technique in the Biomedical Field

The sol-gel synthesis method can be applied for the development of stem cell therapies, especially for the application of lineage-specific cell differentiation inducers, making it very interesting for therapeutic tissue-engineering applications. Examples of this use of the sol-gel synthesis process were published by Robert C. Bielby et al. [94]. They used soluble extracts prepared from 58S bioactive sol-gel glass to enhance lineage-specific differentiation in murine embryonic stem cells into osteogenic cells. They found that the effect of the sol-gel bioactive glass on cell differentiation was dose-dependent, increasing the cell differentiation with higher bioactive glass concentrations, demonstrating the capacity of this inorganic bioactive material to stimulate differentiation of embryonic stem cells into a lineage with therapeutic potential in tissue-engineering applications. Another example was published by Sun-Ae Oh et al. [73]. They used sol-gel bioactive glass granules, obtained from tetraethyl orthosilicate and calcium nitrate tetrahydrate, and doped them with different Zn concentrations to evaluate the effect of these granules in the cell growth and the osteogenic differentiation of mesenchymal stem cells. They found that the zinc addition to the bioactive sol-gel glass increased the growth and the osteogenic differentiation of the stem cells compared with the effect of the Zn-free bioactive glass. Thus, Zn addition to bioactive glass obtained by the sol-gel method may be useful in the development of biomaterials for bone tissue engineering. More recent research published by L. Mosqueira et al. [95] assesses the ability of strontium-releasing sol-gel bioactive glass spheres to stimulate the osteogenic differentiation in osteoporotic bone marrow mesenchymal stem cells. They reported that the Sr-doped bioactive glass particles showed controlled ion release capacity and the stimulation of differentiation potential of bone marrow mesenchymal stem cells from osteoporotic rats due to Sr release from the bioactive sol-gel glass.

Another interesting advancement is the development of stimuli-responsive sol-gel systems that can interact with different physiological parameters, such as pH or temperature, reacting to changes in these parameters. J. F. Mano [96] reported some examples of temperature- and pH stimuli-responsive hydrogels used for biomedical applications such as tissue engineering, drug delivery systems, or biological sensors. Thus, changes in pH or temperature can trigger the release of drugs loaded into stimuli-responsive sol-gel particles, modulating the release rate to adapt it to the actual necessities of the organism.

Finally, sol-gel biohybrid composite materials are innovative tools developed for different uses in the biomedical field, for example, to be used as delivery systems for different molecules such as drugs, growth factors, or cell differentiation inducers. In their research, C. Carrasquilla et al. [97] reported the generation of sol-gel derived biohybrid materials incorporating long-chain DNA aptamers and demonstrated that the aptamers entrapped in the sol-gel matrix can interact with high molecular weight targets such as proteins, making them interesting for molecular recognition systems.

## 7. The Sol-Gel Process in the Biocompatibility Improvement of Magnesium Alloys

### 7.1. General Considerations

The research on different magnesium alloys as biomaterials is widespread in the biomedical field [98,99,100,101]. Magnesium and its alloys present several properties that make them very interesting for the development of biomaterials. Magnesium is a light material that presents the lowest weight/resistance ratio between the structural metals, and a low elastic modulus value that is close to that of living bone tissue, making magnesium and its alloys interesting for developing bone fracture treatment elements. Magnesium and its alloys are bioresorbable materials. Therefore, they could be integrated into the group of second-generation biomaterials. Additionally, magnesium is an essential element in the osteogenesis and angiogenesis processes [102]. Furthermore, the Mg^2+^ ion, which is generated during magnesium corrosion, is involved in several physiological processes and its presence in the blood at a specific level is very important. For example, the low presence of Mg^2+^ in the blood, the phenomenon that is known as hypomagnesemia, is associated with different pathologies such as type 2 diabetes mellitus, osteoporosis, asthma, or vascular disorders [103]. For all these reasons, magnesium and its alloys are very interesting to be used as biomaterials for different purposes such as orthopaedic implants or coronary stents.

However, all these impressive properties of magnesium and its alloys are overshadowed by their main drawback: magnesium is a highly reactive element prone to corroding that produces hydrogen at a high rate. During the corrosion process of magnesium in aqueous media, the hydrogen accumulates around the living tissues surrounding a magnesium-based implant and alters the environment, promoting changes in pH and other factors that could lead to a low implant-tissue attachment, damage to the living tissues, and would end up with the failure of the implant, making necessary a new surgery to extract it from the body of the patient.

Therefore, it is necessary to apply different processes to the implant to control the degradation rate of the magnesium implants and to decrease the amount of hydrogen to a level that is not harmful to the patient, making magnesium-based alloys more biocompatible and increasing their possible usage as biomaterials.

One strategy to reduce the corrosion rate in magnesium implants implies the reduction of impurities such as iron, copper, or nickel that can be present in magnesium samples. These elements form galvanic couples in the alloy, and their presence, even in a low concentration, has shown to directly influence the low corrosion resistance of magnesium [104,105,106]. Therefore, by controlling the processes to obtain ultrapure magnesium implants, the corrosion rate can be reduced.

**Figure 22:**
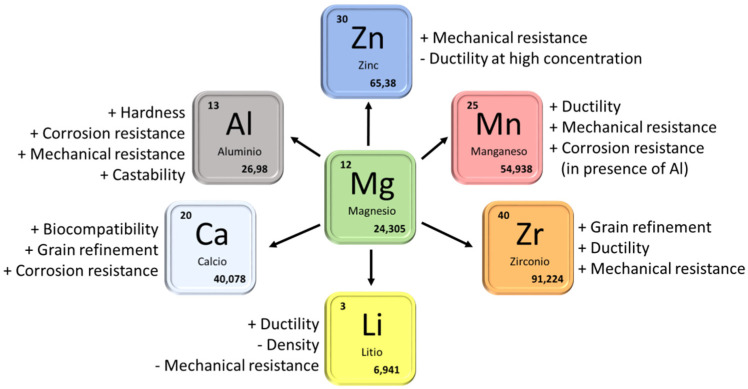
Main elements used for the generation of magnesium alloys with interest in the biomedical field and their effect on the properties of the alloy. Adapted from the information provided by F. Witte et al. [107] and M. Esmaily et al. [108].

The addition of alloying elements is another strategy for improving the properties of magnesium as a biomaterial. Figure 22 shows a resume of the main alloying elements present in magnesium alloys with interest in the biomaterials field and their influence on the properties of the alloy. There are different biphasic alloy systems based on magnesium that are usually presented as potential biomaterials in the scientific literature [107,108]. The most important ones are the Mg-Al, Mg-Ca, Mg-Zn, and Mg Rare Earths alloy systems. Table 3 shows the composition of some of the most interesting magnesium alloys in the biomedical field.

Among all the different strategies that can be followed to achieve an improvement of biocompatibility for magnesium alloys, the application of coatings is very extended [109,110,111,112,113]. By applying coatings on the surface of a biodegradable material, it is possible to control the degradation rate of the implant, slowing down the release rate of the corrosion by-products generated during the biodegradation process, such as hydrogen in the case of magnesium alloys, and increasing the durability of the implants to guarantee that the treatment time is long enough to let the damaged tissues be healed before the loss of mechanical integrity of the implant. As previously exposed, the sol-gel process can play an important role as a synthesis method to generate coatings that can be applied to magnesium alloys to control their degradation rate.

### 7.2. Sol-Gel Coatings on Mg-Al Alloys

The Mg-Al systems are commonly used because the addition of Al improves the mechanical properties and the corrosion resistance of the alloy, which is the most important concern that magnesium presents. The most used Mg-Al alloys are the AM60B, AZ31, AZ60, AZ80, and AZ91D. However, aluminum is known for being neurotoxic at certain concentrations in the body; therefore, their presence must be reduced to a minimum. Thus, AZ31 is one of the most interesting Mg-Al alloys for biomedical applications due to its reduced Al content. Despite the presence of Al increasing the corrosion resistance of these alloys, different surface treatments such as sol-gel coatings are usually necessary to control the degradation rate of these alloys if they are intended to be used as biomaterials.

**Figure 23:**
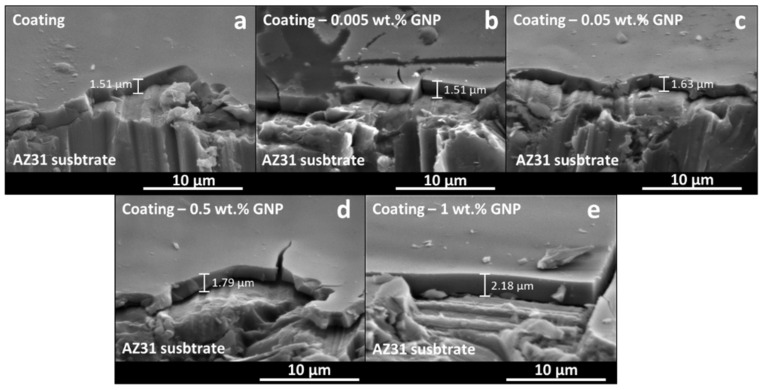
Adapted with permission from J.P. Fernández-Hernán et al. [113]. Copyright 2021 Elsevier. License number 5318820927306. Sol-gel coatings deposited on AZ31 alloy substrates. (**a**) Sol-gel without nanocharges, (**b**) Sol-gel + 0.005 wt.% COOH-GNPs, (**c**) Sol-gel + 0.05 wt.% COOH-GNPs, (**d**) Sol-gel + 0.5 wt.% COOH-GNPs, and (**e**) Sol-gel + 1 wt.% COOH-GNPs.

J.P. Fernández-Hernán et al. [113] developed sol-gel coatings doped with different concentrations of COOH- functionalized graphene nanoplatelets and deposited them on the surface of AZ31 magnesium alloy substrates (Figure 23). They studied the in vitro biocorrosion of the coated samples using Hanks’ solution as electrolyte and found that the lowest concentrations of COOH-GNPs (0.005 and 0.05 wt.%) improved the behavior of the sol-gel coatings against corrosion. Moreover, the biocompatibility of the doped sol-gel coatings was assessed using the MC3T3-E1 preosteoblastic cell line. All the studied conditions let the cell proliferation on the surface of the samples. However, lower cell adhesion was found on the surface of the bare AZ31 substrate than on the surface of the substrates coated with the doped sol-gel coatings.

S. Nezamdoust et al. [114] developed different sol-gel coatings to control the degradation rate of AM60B samples. To generate the sol-gels, they used different precursors such as tetraethoxysilane, 3-glycidyloxypropyl-trimethoxysilane and phenyl-trimethoxysilane in combination with a Ti-Zr conversion coating as pre-treatment to improve the adherence of the sol-gel coating to the substrate, achieving a good improvement in the corrosion behavior of the coated samples immersed in 0.05 M NaCl solution. In other research, they generated composite coatings based on a cerium–vanadium conversion layer covered with a defect-free sol-gel coating generated from tetraethyl orthosilicate and (3-glycidoxypropyl)trimethoxysilane [115]. In that case, the sol-gel coating managed to cover the defects of the previously generated Ce–V conversion layer, achieving good corrosion resistance when the samples were immersed in Harrison’s solution.

One of the most powerful features of the sol-gel synthesis process is the possibility to add different nanoparticles and molecules to the sol to provide improved anticorrosion properties to the final coating. In this regard, the addition of hydroxyl functionalized multiwall carbon nanotubes (OH-MWCNT) showed to increase the protective effect of sol-gel coatings generated from poly-trimethoxyphenylsilane and deposited on AM60B magnesium substrates, in comparison with sol-gel coatings without OH-MWCNT, which showed cracks and defects [116]. Other nanoparticles such as hydroxylated nano-diamond (HND) particles [117] and oxidized fullerene (OF) nanoparticles [118] have been successfully incorporated into sol-gel coatings deposited on Mg-Al alloys, increasing the anticorrosion properties of the coatings.

Kai Huang et al. [119] developed mesoporous bioactive glass coatings with a molar composition of 60% SiO_2_, 36% CaO, and 4% P_2_O_5_ and deposited them on AZ31 alloy substrates by dip-coating. The biocorrosion and bioactivity were assessed in vitro using simulated body fluid (SBF) as the testing medium, proving that these coatings can provide effective biocorrosion protection and promote the generation of HAp on their surfaces at low immersion times in SBF, which is an indicator of bioactivity. A.F. Galio et al. [120] developed doped hybrid sol-gel coatings and deposited them on AZ31 alloy substrates. They used 3-glycidoxypropyltrimethoxysilane and zirconium (IV) tetrapropoxide as the sol-gel precursors, and 8-hydroxyquinoline as the corrosion inhibitor loaded in the sol-gel coatings. The corrosion protection of these coatings was assessed by immersing them in a 0.005 M NaCl solution. They found that the presence of the corrosion inhibitor enhanced the corrosion protection without compromising the barrier effect of the sol-gel coating. Thus, the doped hybrid sol-gel coatings were able to prevent corrosion on the samples after two-week immersion in such an aggressive solution.

The combination of the sol-gel synthesis with any coating method provides a powerful tool to generate multifunctional coatings composed of several layers with different functionalities such as corrosion protection, cellular adhesion promotion, and antibacterial effects, among others. In this regard, Hui Tang et al. [121] used the sol-gel method to generate a bi-layer coating system on AZ31 alloy substrates. These coating systems consisted of an inner TiO_2_ coating and an outer calcium phosphate coating. They also generated monolayer titania and calcium phosphate coatings. The behavior of the coatings was assessed by immersion in SBF. The hydrogen evolution and the electrochemical tests revealed that the multi-layered system achieved the best anticorrosion protection. It is important to notice that, in addition to the corrosion protection, mainly provided by the inner titania layer, the presence of an outer calcium phosphate layer could promote the adhesion and growth of bone cells, which could be very interesting to improve the biocompatibility and osseointegration of implants made from magnesium and coated with these multifunctional coatings.

Although the AZ31 alloy is the most interesting for biological applications among the magnesium alloys of the AZ family due to its low aluminum concentration, other magnesium alloys of this family are commonly used and their biocorrosion behavior is usually improved by the application of sol-gel coatings on their surface. Thus, Y. Su et al. [111] worked with the AZ60 alloy, applying a hydroxyapatite coating generated by sol-gel on a calcium phosphate conversion coating previously formed on the AZ60 substrate. The sol-gel hydroxyapatite coating was supposed to seal the cracks and defects of the calcium phosphate coating, in an attempt to achieve a better barrier effect of the multilayer coating and thus improve the corrosion behavior and the surface biomineralization capability of this alloy. Their results showed that the application of the sol-gel hydroxyapatite coating managed to significantly improve the in vitro corrosion of the AZ60 substrates coated only with the calcium phosphate conversion layer. A similar approximation was obtained by L. Pezzato et al. [122]; they worked with AZ80 alloy substrates over which plasma electrolytic oxidation (PEO) porous coatings were generated. They applied a sol-gel coating to seal the pores of the previously applied PEO coating, improving the barrier effect of the coating, the sol-gel coating was generated from tetraethyl orthosilicate and methyl-triethoxysilane, and the samples were coated by dip-coating. The presence of the sol-gel coating was demonstrated to improve the corrosion behavior when compared with the monolayer PEO coating, due to the capacity of the sol-gel to penetrate and seal the pores of the PEO coating, improving the barrier effect.

Another commonly used magnesium alloy of the AZ family is AZ91. However, due to its high aluminum content, the use of this alloy for biomedical applications can be controversial. That is the reason why the use of this alloy is more suitable for other purposes in the aerospace or transport industries. However, examples can be found in the literature where sol-gel coatings are applied to this alloy to improve its biodegradability properties [123].

### 7.3. Sol-Gel Coatings on Mg-Zn, Mg-Ca, and Mg-Rare Earth

As previously explained, the presence of a high concentration of aluminum as an alloying element is controversial for the generation of magnesium alloys intended to be used in the biomedical industry. Thus, other magnesium alloys without aluminum can be used to manufacture implants and elements for the treatment of bone fractures. The lack of aluminum in the alloy decreases the resistance to biocorrosion. However, the use of sol-gel coatings on these alloys can help to achieve adequate control of the degradation rate. The most important elements used to generate aluminum-free magnesium alloys are zinc and calcium, both of which are naturally present in the organism, and Zn^2+^ and Ca^2+^ cations take part in different physiological and biological regulation processes [124]. The presence of Zn as an alloying element led to the generation of intermetallic compounds and provides a gain refinement to the alloy. The addition to Ca also provides a grain refinement and is an interesting alloy element for bone regeneration biomaterials. 

**Figure 24:**
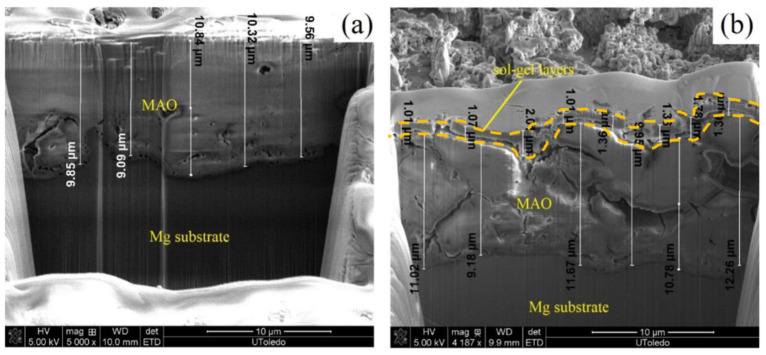
Adapted with permission from H. Ibrahim et al. [127]. Copyright 2019 Elsevier. License number 5318830570042. SEM cross-section images of (**a**) PEO coating and (**b**) PEO + sol-gel coating.

Regarding Mg–Zn alloys, L.M Rueda et al. [125] designed hybrid sol-gel coatings to improve the in vitro corrosion of ZE41 alloy substrates. The main alloying element, in this case, is Zn, but also rare earth such as La, Ce, and Ga can be found. The sol-gel precursors were tetraethyl orthosilicate and (3-glycidoxypropyl)trimethoxysilane and the coatings were generated on the ZE41 substrates by dip-coating. The sol-gels with different ageing times, from 2 h to 5 days, showed good corrosion protection when the coated samples were immersed in SBF at 37 °C. In their research, A. Chmielewska et al. [126] developed TiO_2_ sol-gel coatings with HAp/β-TCP nanoparticles and deposited them on MAO (micro-arc oxidation)-coated Mg—1.2, Zn—0.5, Ca—0.5 Mn alloy coupons. The sol-gel coating covered and filled the pores of the previously grown MAO coating. They carried out cytocompatibility tests using the L929 murine fibroblasts cell line and found that the presence of sol-gel coatings provided a statistically significant improvement regarding cell viability compared with the As-cast and MAO coated samples. H. Ibrahim et al. [127] generated Mg—1.2Zn—0.5Ca—0.5Mn alloy samples and treated them with PEO (plasma electrolytic oxidation) coatings combined with TiO_2_ sol-gel coatings doped with HAp/β-TCP nanoparticles (Figure 24). 

The sol-gel coating covered the pores of the underlying PEO coating, resulting in a much lower and uniform corrosion. The presence of these PEO/sol-gel coatings significantly decreased the loss of mechanical strength of the coated samples, adjusting the degradation rate to the expected time necessary for the completion of the healing process in a bone fracture.

In the case of Mg–Ca alloys for biomedical applications, M. Li et al. [128] developed TiO_2_ coatings by the sol-gel synthesis process, using tetrabutylorthotitanate (C_16_H_36_TiO_4_) as a Ti precursor, and deposited them on Mg—1.0Ca alloy substrates by dip-coating. They evaluated the corrosion behavior of the different conditions (bare and TiO_2_-coated substrates) using electrochemical measurements and immersion test in simulated body fluid (SBF), finding that the presence of the TiO_2_ sol-gel coating led to a decrease of the current density in almost three orders of magnitude compared with the bare Mg—1.0Ca alloy substrate.

Regarding Mg rare earth alloys, A. Roy et al. [129] developed Calcium phosphate (CaP) and Si-containing sol-gel coatings, using calcium nitrate, phosphorus pentoxide, and tetraethyl orthosilicate as precursors, and deposited them on Mg4Y alloy substrates. They carried out in vitro biodegradation tests by immersion of the coated and bares substrates in DMEM medium, and cytocompatibility tests seeding MC3T3-E1 osteoblasts on the different samples. They found that the presence of the sol-gel coatings improved the cytocompatibility compared with the uncoated Mg4Y alloy surfaces.

## 8. Concluding Remarks and Outlook

The sol-gel synthesis method shows powerful potential to generate materials with interest in the biomedical field for several applications exposed in this review. Sol-gel coatings can be generated and applied on implants made out of metallic or ceramic materials to control the biocorrosion resistance or the biocompatibility of the implants. Additionally, scaffolds and matrices can be generated and used in tissue engineering applications, for example promoting cell growth and differentiation. Finally, sol-gel micro- and nanoparticles can be loaded with different substances such as antibiotics and be used as carriers for drug delivery systems.

Compared with other techniques, the main advantage provided by the sol-gel synthesis method is that it is a low-energy and cost-effective method to obtain bioactive materials that can be used in the newest biomedical advances such as tissue engineering or stem cell therapies. Important biomaterials can be obtained using this technique, such as hydroxyapatite or other Ca/P compounds that have been used, for example, to induce the growth of bone cells when they are put in contact with natural bone tissue, making them very interesting to be used for in situ treatment of bone fractures.

The sol-gel technique is a very useful tool for developing cutting-edge technologies such as composite biomaterials or biohybrid materials. Porous gel matrices can be obtained that can be loaded not only with drugs for the development of drug delivery systems but with living cells, in an attempt to generate biohybrid composites that could act as bioartificial organs which, in the future, could be implanted in the body directly in a damaged organ to restore it.

The perspectives of the use of the sol-gel technique in the biomedical field are good as it can be applied for innovative applications in stem cell therapies or for the generation of stimuli-responsive delivery systems, interesting for developing future personalized treatments. Additionally, the possibility to generate biocompatible nanoparticles which can be loaded with drugs allows the development of accurate and minimally invasive tools for the treatment of different diseases. The use of these drug-loaded nanoparticles arouses special interest in future cancer treatments. Thus, despite being a well-known and extensively used technique, the sol-gel synthesis method can play an important role in the development of future biomaterials and therapies.

## Figures and Tables

**Table 1:** Different generations of biomaterials.

Generation	Year	Properties	Biomaterials
First	~1960	Bioinert, Non-toxic.	Stainless steel, Cr-Co-Mo, NiTi, Ti6Al4V.
Second	1970–1990	Bioactive, Biodegradable.	Hydroxyapatite, Bioactive Glass, Magnesium alloys.
Third	~2000	Bioactive, Biodegradable, Cause specific cellular response.	Bioglass^®^, Biosilicates.
Fourth	~2015	Interactions with cellular signals.	Carbon-based materials, Conductive polymers.

**Table 2:** Sol-gel synthesis method applied for the generation and development of biomaterials and tissue engineering devices.

Morphology	Synthesized Material	Applications	References
Coating	Hydroxyapatite (Ca_5_(PO_4_)_3_OH)	Biocompatibility improvement, Osseointegration, Antimicrobial.	[5,19,27,28,35,40,42]
	Bioactive glass (SiO_2_-CaO-P_2_O_5_)	Biocompatibility improvement, Osseointegration.	[19,37,38]
	SiO_2_	Biocompatibility improvement, Biocorrosion protection, Drug delivery.	[30,34,52,66]
	TiO_2_	Biocorrosion protection, Biocompatibility improvement, Osseointegration.	[50,51,65]
	ZnO	Antimicrobial.	[49]
	Nb_2_O_5_	Biocorrosion protection, Biocompatibility improvement.	[21,64]
Particles	Bioactive glass (SiO_2_-CaO-P_2_O_5_)	Biocompatibility improvement.	[79,82]
	Hydroxyapatite (Ca_5_(PO_4_)_3_OH)	Antimicrobial, Drug delivery, Biocompatibility improvement.	[80,81,82,85]
	SiO_2_	Drug delivery.	[70,83]
Matrix	SiO_2_	Drug delivery.	[10,86]
	Alginate/SiO_2_	Drug delivery, Bio-artificial organ.	[88]
	Collagen/SiO_2_	Drug delivery, Bio-artificial organ.	[90]
Scaffold	Bioglass^®^	Tissue engineering, Osseointegration.	[91,92]
	Hydroxyapatite/Polycaprolactone	Tissue engineering, Osseointegration.	[93]

**Table 3:** Composition of different magnesium alloys.

Alloy Elements	Alloy	Composition
Mg–Al	AM60B	Al (5.5–6.5%), Mn (0.24–0.6%), Zn (0.22%) and <0.05% Si, Cu, Fe, Ni
	AZ31	Al (2.5–3.5%), Zn (0.6–1.4%), Mn (0.2%) and <0.1% Si, Cu, Ca, Fe, Ni
	AZ60	Al (5.8–7.2%), Zn (0.4–1.5%), Mn (0.15%) and <0.1% Si, Cu, Ni, Fe
	AZ80	Al (7.8–9.2%), Zn (0.2–0.8%), Mn (0.12%) and <0.1% Si, Cu, Fe, Ni
	AZ91D	Al (8.3–9.7%), Zn (0.35–1%), Mn (≥0.13%) and <0.1% Si, Cu, Fe, Ni
Mg–Zn	ZE41	Zn (3.5–5%), Zr (0.4–1%), Rare Earths (La, Ce, Ga 0.75–1.75%), 0.15% Mn
	ZK30	Zn (3%) and Zr (≥0.45%)
	ZK60	Zn (6%) and Zr (≥0.45%)
	Mg3Zn	Zn (3%)
	Mg2Zn	Zn (2%)
Mg–Ca	Mg–1.0Ca	Ca (1%)
	Mg–0.6Ca	Ca (0.6%)
Mg–Zn–Ca	Mg–0.5Zn–0.3Ca	Zn (0.5%), Ca (0.3%)
Mg–Rare Earths	Mg4Y	Y (4%)

## Data Availability

Not applicable.
